# Assessing anorectal function in patients with recurrent ulcerative colitis

**DOI:** 10.1007/s00384-024-04680-1

**Published:** 2024-07-16

**Authors:** Qiaoyan Wu, Tongyu Li, Fenglian Deng, Xuejie Yao, Xueqin Chen, Qi Jiang, Xiaoyun Ding

**Affiliations:** 1 Department of Gastroenterology, The First Affiliated Hospital of Ningbo University, No. 59, Liuting Street, Zhejiang Province, Ningbo, 315010 China; 2 Ningbo Key Laboratory of Translational Medicine Research On Gastroenterology and Hepatology, No. 59, Liuting Street, Zhejiang Province, Ningbo, 315010 China; 3 Department of Hematology, The First Affiliated Hospital of Ningbo University, No. 59, Liuting Street, Zhejiang Province, Ningbo, 315010 China; 4 Hospital Quality Management Office, The First Affiliated Hospital of Ningbo University, No. 59, Liuting Street, Zhejiang Province, Ningbo, 315010 China

**Keywords:** Ulcerative colitis, Relapse, Anorectal function, Determinants

## Abstract

**Purpose:**

Ulcerative colitis (UC) is an inflammatory bowel disease with an unclear etiology that can lead to irreversible changes in distal colonic function in chronic patients. This study investigated anorectal function in recurrent UC patients and identified influencing factors.

**Methods:**

This prospective study enrolled 33 recurrent UC patients and 40 newly diagnosed patients from January 2019 to December 2022. Data collection included clinical records, scores, and anorectal function assessments. Regression analyses were used to identify factors impacting anorectal function.

**Results:**

Recurrent UC patients had higher baseline CRP and fecal calprotectin levels, increased anxiety and depression, and more severe fecal incontinence. They also had lower BMIs, serum Hb and albumin (ALB) levels, and Inflammatory Bowel Disease Questionnaire scores than did initial-onset UC patients. Multivariate linear regression analysis revealed that long disease duration (coef. − 0.376, *P* < 0.001) and high fecal calprotectin level (coef. − 0.656, *P* < 0.001) independently influenced the initial sensation threshold in recurrent UC patients. Additionally, high fecal calprotectin (coef. − 0.073, *P* = 0.013) and high Zung Self-Rating Anxiety Scale score (coef. − 0.489, *P* = 0.001) were identified as two independent determinants of the defecation volume threshold. For the defecation urgency threshold, the independent factors included high disease duration (coef. − 0.358, *P* = 0.017) and high fecal calprotectin level (coef. − 0.499, *P* = 0.001). Similarly, the sole independent factor identified for the maximum capacity threshold was high fecal calprotectin (coef. − 0.691, *P* = 0.001).

**Conclusion:**

Recurrent UC patients had increased rectal sensitivity and compromised anorectal function, which significantly impacted quality of life. Proactively managing the disease, reducing UC relapses, and addressing anxiety are effective measures for improving anorectal function in these patients.

**Supplementary Information:**

The online version contains supplementary material available at 10.1007/s00384-024-04680-1.

## Introduction

Ulcerative colitis (UC) manifests as a nonspecific inflammatory gastrointestinal ailment, and its etiology has yet to be definitively elucidated [1]. The clinical presentation of UC primarily includes abdominal pain, diarrhea, mucous and purulent bloody stools, and extraintestinal and systemic manifestations [2]. Currently, in European and American nations, the annual incidence rate of UC is approximately 24.3 per 100,000 people [3]. In recent years, concomitant with China’s economic growth and dietary shifts, the incidence of UC in China has been steadily increasing, with a current prevalence rate of 2.63 per 100,000 people. UC has progressively established itself as a common digestive disorder [4].

UC is a chronic, progressive ailment characterized by recurrent and lingering episodes. A longitudinal study spanning over a decade revealed irrevocable alterations in distal colonic function, including rectal-anal dysfunction, anorectal incontinence, and irreversible changes in osmotic pressure, among chronic UC patients [5]. The root cause of these transformations lies in the current treatment focus for UC patients, which predominantly centers on mucosal healing, without addressing the restoration of intestinal function and physiology [6, 7]. This protracted course has a dual impact on patients, both physiologically and psychologically, severely compromising their quality of life and imposing substantial economic burdens [8].

Hence, the objective of this prospective study was to explore changes in rectal and anal function among recurrent UC patients and identify correlated factors contributing to functional impairment in these patients. This endeavor aims to develop more effective strategies for early intervention and treatment, mitigating irreversible damage to normal physiological functions of the intestines. Such an approach holds paramount importance in improving patients’ quality of life and reducing health care costs.

## Materials and methods

### Patients

This study received approval from the Ethics Committee of the First Affiliated Hospital of Ningbo University (2018-R049). All patients were informed of the objectives and methodology of the study and provided written consent. This prospective study included 33 patients with recurrent UC and 40 patients with initial UC who were enrolled from January 2019 to December 2022. Sample size calculations were performed using G*Power 3.1.9.7 statistical power analysis software (https://www.gpower.hhu.de/en.html) and referenced existing literature. Patient groups with around 20 participants were deemed reasonable. The comprehensive inclusion criteria and exclusion criteria are outlined in the Supplementary Methods section.

### Data collection

General information regarding the enrolled patients was collected, including sex, age at UC diagnosis, past medication history, disease extent according to the Montreal classification, disease manifestation based on the Truelove-Witts classification [9], presence of extraintestinal manifestations, and more. Baseline measurements included BMI, ESR, serum CRP, Hb and albumin (ALB) levels, fecal calprotectin levels, Inflammatory Bowel Disease Questionnaire (IBD-Q) score [10], and self-rated anxiety and depression assessed using the Zung Self-Rating Anxiety/Depression Scale (SAS/SDS) score, Cleveland Clinic Fecal Inconsistency (CCFI) score [11], and Ulcerative Colitis Endoscopic Index of Severity (UCEIS) score [12]. Notably, special attention was directed towards rectal inflammation during endoscopic examinations, with endoscopies performed within the preceding three months being deemed sufficient. UCEIS scores were determined by two experienced endoscopists who collaboratively assessed severity.

### Study questionnaires

The IBD-Q questionnaire scale, which utilizes the Italian version of the IBD-Q, was used to assess patients’ quality of life. The IBD-Q consists of 32 questions and 4 subscales, namely, bowel function, systemic function, emotional function, and social function. Each question is scored from 1 (worst condition) to 7 (best condition), yielding a total score ranging from 32 to 224. The SAS/SDS comprises 20 items, each reflecting subjective feelings of anxiety or depression. Questionnaire details are presented in the Supplementary Methods.

### Digital rectal examination

The enrolled patients underwent a digital rectal examination, which was conducted by one of the participating clinicians. During this examination, patients were positioned in the left lateral position with proper illumination. A lubricated, gloved index finger was inserted into the rectum to assess the presence or absence of an external anal sphincter (EAS) defect and to measure resting and incremental squeeze pressure. The investigator determined whether the resting pressure or incremental squeeze pressure was categorized as “normal,” “decreased,” or “absent.” These assessments were carried out by surgical staff specializing in colorectal procedures.

### Rectal and anal function testing

The testing apparatus employed was a high-resolution 24-channel anal and rectal pressure testing device (GAP-24A, Ningbo Maida Medical Instruments Co., Ltd., China), which dynamically recorded the entire movement of the rectum and anus in real time. The system utilized a multichannel pressure measurement lead integrator, significantly reducing both pre- and postexamination preparation and disinfection time, thereby enhancing efficiency. The entire testing procedure can be completed within 15 min. No fasting or intestinal preparation was required on the day of the examination. Before the operation commenced, patients were advised to empty their bladder and bowels. For those experiencing difficulty in defecation, an enema may be administered approximately 1 h before the procedure to assist. During the examination, patients were positioned on their left side in a flexed-knee position. A digital rectal examination was conducted to guide patients in simulating defecation and anal contraction movements. A pressure measurement catheter was then inserted through the anal canal.

### Statistical analysis

Statistical analysis was conducted using SPSS 26.0 software. For parametric data with homogeneity of variance and a normal distribution, the results are presented as the mean ± standard deviation (SD). For nonparametric data or data with unequal variances and skewed distributions, the results are represented as medians along with their interquartile ranges (IQRs). Categorical data are presented as counts and percentages. Parametric data were analyzed using t tests or rank-sum tests, and categorical data were analyzed using chi-square tests or Fisher’s exact probability test, as appropriate. Single and multiple linear regression analyses were performed to identify factors influencing changes in anorectal function in patients with chronic recurrent UC. Assumptions for linear regression were confirmed through scatter plots for linearity, the Durbin-Watson test for independence, Cook’s distance for outliers, standardized residual histograms and P-P plots for normality, and residual plots for homoscedasticity. Multicollinearity was ruled out using correlation coefficients, variance inflation factors (VIF), and tolerance values. *P* < 0.05 was considered to indicate statistical significance.

## Results

### Clinical characteristics of primary and recurrent UC patients

**Fig. 1:**
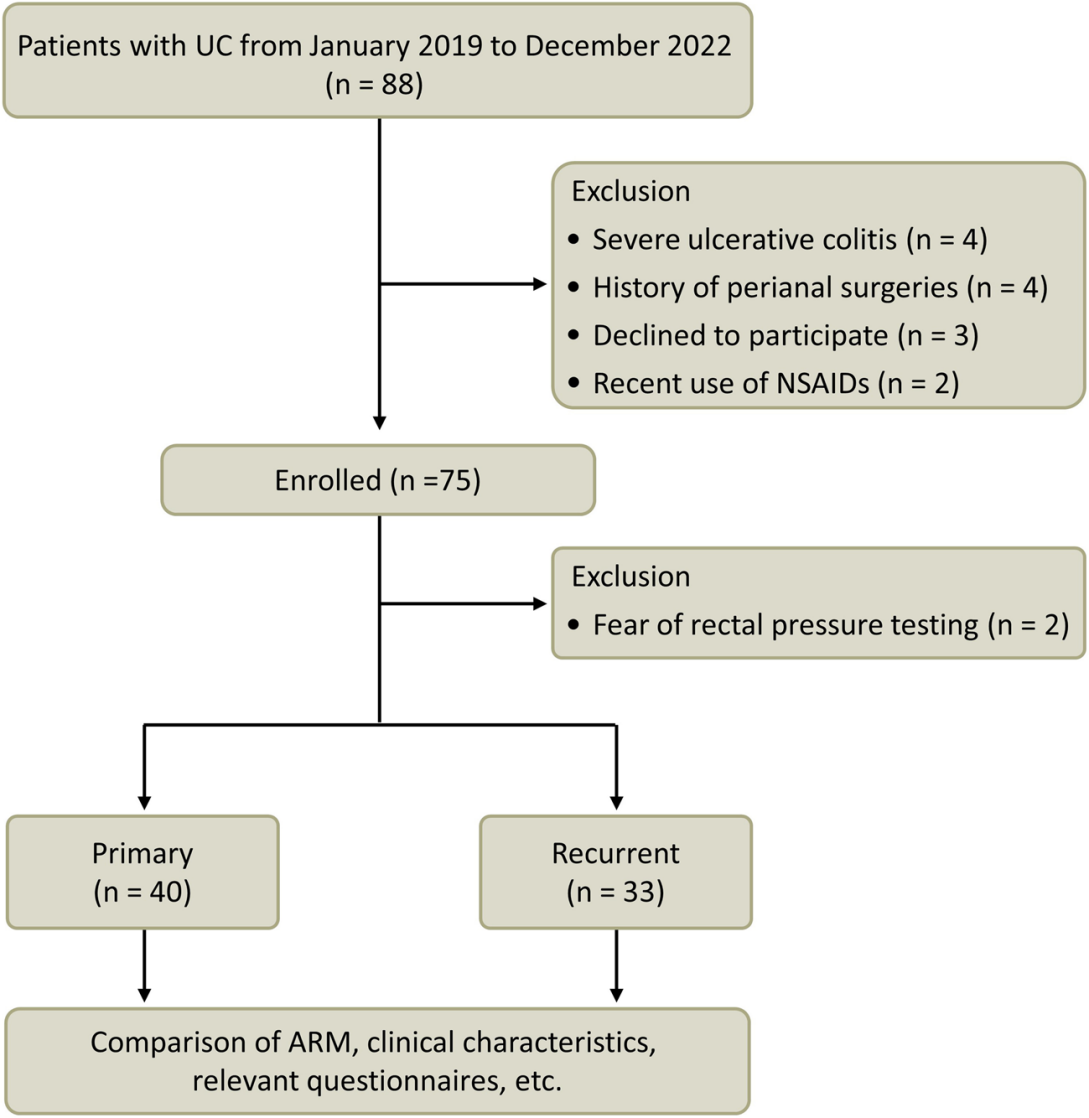
A flowchart showing the study design

During the period from January 2019 to December 2022, a total of 33 recurrent UC patients and 40 initial-onset UC patients were included in the study (Fig. 1). Initial-onset UC patients received no treatment before enrollment. For those experiencing recurrent UC, therapeutic interventions included aminosalicylates (27, 67.5%), immunomodulators (5, 12.5%), biologics (5, 12.5%), and topical mesalazine (3, 7.5%), spanning an average duration of 18.0 ± 2.0 months. The findings revealed no statistically significant differences between the two groups in terms of age, sex, baseline ESR, disease location, disease activity, or UCEIS scores (all *P* > 0.05). However, patients with recurrent UC presented a longer disease course, higher baseline levels of CRP and calprotectin, more pronounced anxiety and depression, greater severity of fecal incontinence, and lower BMI, Hb, ALB, and IBD-Q scores than did those with newly diagnosed UC (all *P* < 0.05; Table 1).

**Table 1:** Comparative clinical characteristics of recurrent and primary UC patients

Variables	Recurrent (*n* = 33)	Primary (*n* = 40)	*P*-value
Male sex (*n*, %)	18 (54.5)	22 (55.0)	0.969
Age (years, mean, SD)	41.9 (14.9)	45.5 (14.0)	0.290
BMI (kg/m^2^, mean, SD)	20.6 (1.8)	23.4 (1.6)	< 0.001
CRP (mg/L, median, IQR)	5 (22.6)	1 (6.6)	0.019
ESR (mm/h, median, IQR)	9 (23.0)	8.5 (17.0)	0.443
Hb (g/L, mean, SD)	121.2 (10.7)	132.6 (17.1)	0.001
ALB (g/L, mean, SD)	41.4 (4.8)	43.6 (4.2)	0.044
Fecal calprotectin (µg/g, mean, SD)	164.1 (43.3)	110.5 (18.4)	< 0.001
Disease duration (years, median, IQR)	5.1 (4.5)	2.4 (1.5)	< 0.001
Disease location (*n*, %)			0.062
E1	15 (45.5)	18 (45.0)	
E2	16 (48.5)	12 (30.0)	
E3	2 (6.1)	10 (25.0)	
Clinical disease activity (*n*, %)			0.135
Mild	23 (69.7)	21 (52.5)	
Moderate	10 (30.3)	19 (47.5)	
IBD-Q score (mean, SD)	165.3 ± 13.2	185.6 ± 11.5	< 0.001
SAS score (mean, SD)	66.2 ± 7.9	54.9 ± 7.5	< 0.001
SDS score (mean, SD)	57.8 ± 7.8	47.2 ± 8.0	< 0.001
CCFI score (mean, SD)	9.5 ± 2.1	7.8 ± 2.9	0.008
UCEIS score (*n*, %)			0.164
Mild	29 (87.8)	30 (75.0)	
Moderate to severe	4 (12.1)	10 (25.0)	
Resting pressure (*n*, %)			
Absent	1 (3.0)	1 (2.5)	1.000
Decreased	2 (6.1)	2 (5.0)	1.000
Normal	30 (90.9)	37 (92.5)	1.000
Squeeze pressure (*n*, %)			
Absent	1 (3.0)	1 (2.5)	1.000
Decreased	3 (9.1)	2 (5.0)	0.653
Normal	29 (87.9)	37 (92.5)	0.694
EAS defect	1 (3.0)	1 (2.5)	1.000

### Rectoanal function assessment in primary and recurrent UC patients

**Fig. 2:**
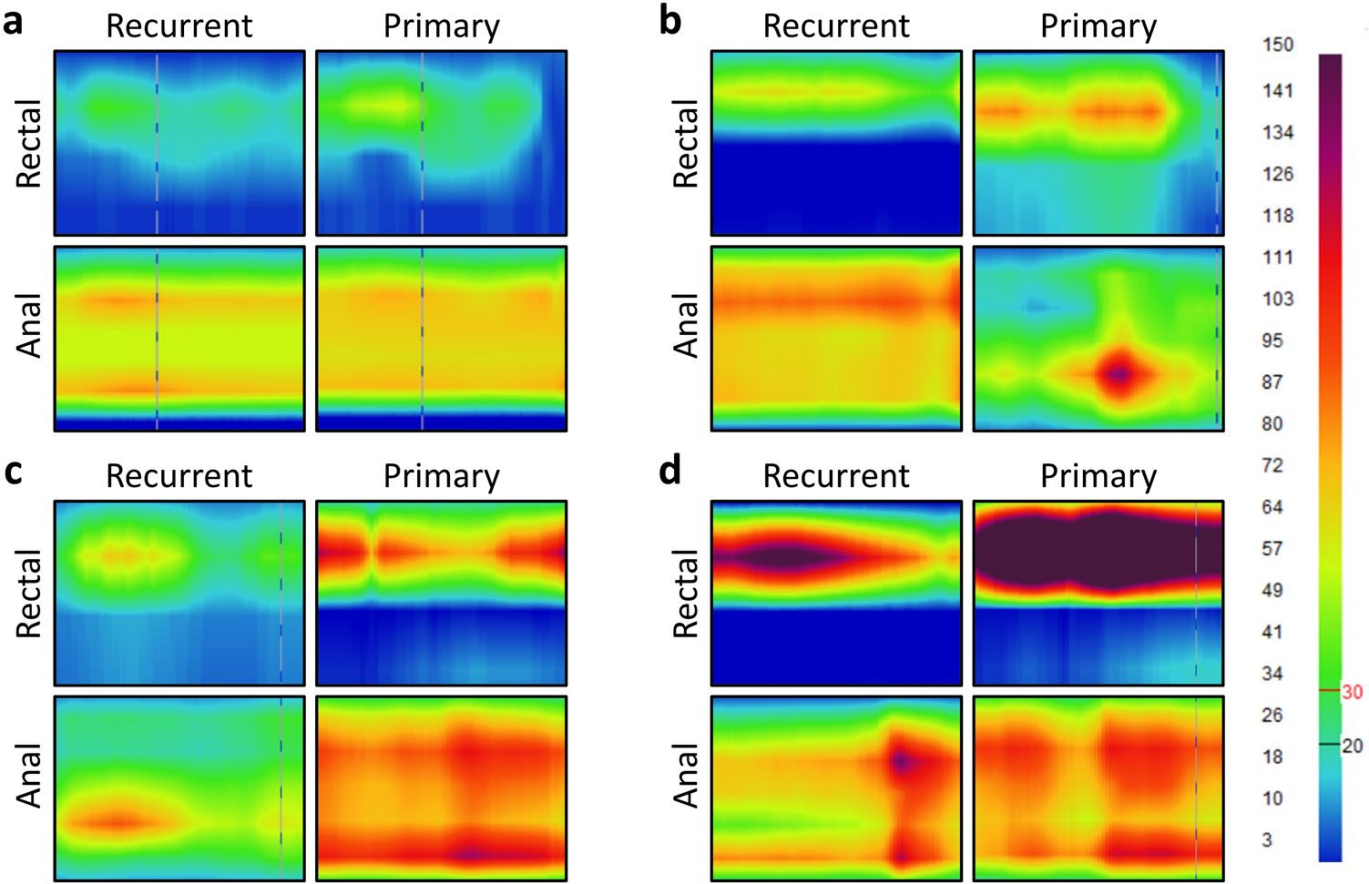
Representative color contour traces reflecting both rectal and anal sphincter pressures for recurrent and primary UC patients, demonstrating the initial sensation threshold (**a**), defecation volume threshold (**b**), defecation urgency threshold (**c**), and maximum capacity threshold (**d**)

In this study, patients with recurrent UC had lower initial sensation thresholds, defecation volume thresholds, defecation urgency thresholds, and maximum capacity thresholds than their newly diagnosed counterparts (all *P* < 0.05). Concurrently, the maximum squeeze pressure (MSP) in recurrent UC patients was inferior to that in primary UC patients (*P* < 0.05). However, 9.1% (3/33) of patients in the recurrent UC group had an MSP < 90 mmHg, which was less than the 15% (6/40) of patients in the primary UC group. However, this difference was not statistically significant (*P* > 0.05). Additionally, while the mean minimum volume required to elicit the RAIR was not significantly different between the groups (*P* > 0.05), both cohorts were susceptible to RAIR induction. Furthermore, paradoxical contractions were present in approximately 50% of patients in both groups, but the difference between the groups did not reach statistical significance (*P* > 0.05) (Fig. 2; Table 2).

**Table 2:** Anorectal manometry: recurrent and primary UC patients

Variables	Recurrent (*n* = 33)	Primary (*n* = 40)	*P*-value
Rectal resting pressure (mmHg, mean, SD)	50.3 ± 7.4	53.1 ± 5.0	0.057
Anal sphincter resting pressure (mmHg, mean, SD)	67.7 ± 14.3	73.0 ± 16.7	0.150
Rectal pressure at simulated defecation (mmHg, mean, SD)	50.6 ± 20.0	55.1 ± 19.6	0.619
Initial sensation threshold (mL, mean, SD)	22.6 ± 5.6	36.5 ± 9.6	< 0.01
Defecation volume threshold (mL, mean, SD)	43.0 ± 8.6	71.4 ± 16.6	< 0.01
Defecation urgency threshold (mL, mean, SD)	72.6 ± 12.0	98.5 ± 21.3	< 0.01
Maximum capacity threshold (mL, mean, SD)	93.9 ± 15.1	132.5 ± 23.6	< 0.01
MSP (mmHg, mean, SD)	102.5 ± 13.7	117.3 ± 31.3	0.014
Rectal compliance (mmHg, mean, SD)	2.7 ± 0.9	2.3 ± 0.9	0.063
Minimum volume to elicit RAIR (mL, mean, SD)	10.3 ± 1.7	11.0 ± 3.0	0.247
Paradoxical contraction (*n*, %)	20.0 (60.6)	19.0 (47.5)	0.264

### Linear regression analysis of rectal sensitivity in recurrent UC patients

Univariate linear regression analysis indicated a significant association between the initial sensation threshold of patients with recurrent UC and disease duration (*P* < 0.001), ESR (*P* = 0.022), fecal calprotectin level (*P* < 0.001), IBD-Q score (*P* < 0.001), SAS score (*P* = 0.003), SDS score (*P* = 0.002), and CCFI score (*P* = 0.026). The defecation volume threshold in these patients was significantly correlated with disease duration (*P* < 0.001), fecal calprotectin level (*P* = 0.006), IBD-Q score (*P* = 0.007), SAS score (*P* = 0.002), SDS score (*P* = 0.001), CCFI score (*P* = 0.015), and UCEIS score (*P* = 0.043). Furthermore, the defecation discomfort threshold was significantly correlated with disease duration (*P* < 0.001), fecal calprotectin level (*P* < 0.001), IBD-Q score (*P* = 0.005), SAS score (*P* = 0.002), SDS score (*P* = 0.001), CCFI score (*P* = 0.040), and UCEIS score (*P* = 0.028). Finally, the maximum capacity thresholds of these patients were significantly correlated with disease duration (*P* = 0.009), fecal calprotectin level (*P* < 0.001), IBD-Q score (*P* = 0.027), and CCFI score (*P* = 0.041) (Table 3). Multivariate linear regression analysis revealed that *long* disease duration (coef. − 0.376, *P* < 0.001) and high fecal calprotectin level (coef. − 0.656, *P* < 0.001) independently influenced the initial sensation threshold in recurrent UC patients. Additionally, high fecal calprotectin (coef. − 0.073, *P* = 0.013) and high SAS score (coef. − 0.489, *P* = 0.001) were identified as two independent determinants of the defecation volume threshold. For the defecation urgency threshold, the independent factors included high disease duration (coef. − 0.358, *P* = 0.017) and high fecal calprotectin level (coef. − 0.499, *P* = 0.001). Similarly, the sole independent factor identified for the maximum capacity threshold was high fecal calprotectin (coef. − 0.691, *P* = 0.001) (Table 4).

**Table 3:** Univariate linear regression analysis on anorectal manometry data in patients with recurrent UC

Variables	Initial sensation threshold			Defecation volume threshold			Defecation urgency threshold			Maximum capacity threshold		
	Coef. (95% CI)	*P*	*R*^2^	Coef. (95% CI)	*P*	*R*^2^	Coef. (95% CI)	*P*	*R*^2^	Coef. (95% CI)	*P*	*R*^2^
Female*	− 0.15 (− 5.68, 2.35)	0.404	0.023	− 0.00 (− 6.26, 6.15)	0.996	0.000	− 0.10 (− 10.34, 5.79)	0.569	0.011	− 0.19 (− 16.06, 5.17)	0.304	0.034
Age	− 0.33 (− 0.25, 0.01)	0.061	0.109	− 0.33 (− 0.39, 0.01)	0.058	0.111	− 0.24 (− 0.45, 0.09)	0.181	0.057	− 0.13 (− 0.50, 0.23)	0.466	0.017
BMI	0.05 (− 1.00, 1.32)	0.781	0.003	− 0.01 (− 1.81, 1.74)	0.967	0.000	0.04 (− 2.06, 2.58)	0.229	0.002	0.19 (− 1.44, 4.55)	0.297	0.035
hs-CRP	− 0.04 (− 0.12, 0.09)	0.811	0.002	− 0.19 (− 0.24, 0.07)	0.289	0.036	− 0.11 (− 0.27, 0.15)	0.546	0.012	0.01 (− 0.27, 0.29)	0.955	0.000
ESR	− 0.39 (− 0.24, − 0.02)	0.022	0.158	− 0.19 (− 0.27,0.09)	0.301	0.034	− 0.14 (− 0.33, 0.15)	0.448	0.019	− 0.07 (− 0.38, 0.25)	0.682	0.005
Hb	0.31 (− 0.01, 0.22)	0.082	0.097	0.25 (− 0.06, 0.40)	0.174	0.062	0.31 (− 0.03, 0.44)	0.086	0.095	0.21 (− 0.13, − 0.50)	0.241	0.045
ALB	0.19 (− 0.24, 0.75)	0.302	0.035	0.03 (− 0.71, 0.81)	0.893	0.005	0.17 (− 0.55, 1.44)	0.366	0.027	0.20 (− 0.57, 2.01)	0.265	0.041
Fecal calprotectin	− 0.85 (− 0.14, − 0.09)	< 0.001	0.719	− 0.47 (− 0.16, − 0.03)	0.006	0.562	− 0.58 (− 0.23, − 0.07)	< 0.001	0.331	− 0.69 (− 0.32, − 0.14)	< 0.001	0.474
Disease duration	− 0.71 (− 1.20, − 0.56)	< 0.001	0.489	− 0.64 (− 1.74, − 0.67)	< 0.001	0.385	0.61 (− 2.22, − 0.78)	< 0.001	0.347	− 0.45 (− 0.21, − 0.39)	0.009	0.174
Disease location			0.072			0.027			0.018			0.004
E2 vs. E1	− 0.28 (− 7.16, 1.03)	0.137		− 0.14 (− 8.78, 4.03)	0.455		− 0.12 (− 11.14, 5.68)	0.512		− 0.01 (− 11.68, 10.88)	0.943	
E3 vs. E1	− 0.07 (− 10.11, 6.98)	0.711		− 0.12 (− 17.74, 14.90)	0.509		− 0.09 (− 21.60, 13.48)	0.640		− 0.07 (− 27.60, 19.48)	0.727	
Disease activity			0.061			0.099			0.092			0.043
Mild	Reference			Reference			Reference			Reference		
Moderate	− 0.25 (− 7.24, 1.29)	0.164		− 0.32 (− 12.61, 0.60)	0.074		− 0.30 (− 15.65, 1.08)	0.086		− 0.21 (− 18.10, 4.79)	0.245	
IBD-Q score	0.63 (0.15, 0.39)	< 0.001	0.280	0.48 (0.10, 0.52)	0.005	0.227	0.48 (0.13, 0.68)	0.005	0.228	0.38 (0.05, 0.82)	0.027	0.148
SAS score	− 0.50 (− 0.58, − 0.13)	0.003	0.310	− 0.51 (− 0.90, − 0.22)	0.002	0.264	− 0.51 (− 1.17, − 0.28)	0.002	0.264	− 0.19 (− 1.03, 0.31)	0.278	0.038
SDS score	− 0.53 (− 0.60, − 0.15)	0.002	0.339	− 0.54 (− 0.94, − 0.26)	0.001	0.293	− 0.57 (− 1.25, − 0.38)	0.001	0.319	− 0.34 (− 1.316, 0.02)	0.056	0.113
CCFI score	− 0.39 (− 1.93, − 0.13)	0.026	0.182	− 0.42 (− 3.06, − 0.36)	0.015	0.178	− 0.36 (− 3.72, − 0.09)	0.040	0.129	− 0.36 (− 4.86, − 0.10)	0.041	0.128
UCEIS score			0.117			0.125			0.146			0.048
Mild	Reference			Reference			Reference			Reference		
Moderate to severe	− 0.34 (− 11.60, 0.05)	0.052		− 0.35 (− 17.99, − 0.28)	0.043		− 0.38 (− 24.32, − 1.46)	0.028		− 0.22 (− 25.91, 6.25)	0.222	

Dependent variables: Initial sensation threshold,defecation volume threshold, defecation urgency threshold, and maximum capacity threshold

^*^Relative to male

^†^Results were from simple linear regression models. Coef. represents standardized β coefficient

**Table 4:** Multivariate linear regression analysis on anorectal manometry data in patients with recurrent UC

Independent variables	Dependent variable	
	Coef. (95% CI)	*P*
Initial sensation threshold		
ESR	0.030	0.837
Fecal calprotectin	− 0.656 (− 0.109, − 0.061)	< 0.001
Disease duration	− 0.376 (− 0.691, − 0.241)	< 0.001
IBD-Q	− 0.006	0.956
SAS	0.089	0.403
SDS	− 0.062	0.553
CCFI	0.036	0.681
*R*^2^	0.824	
Durbin-Watson	1.543	
Defecation volume threshold		
Fecal calprotectin	− 0.073 (− 0.129, − 0.017)	0.013
Disease duration	− 0.106	0.555
IBD-Q	0.057	0.802
SAS	− 0.489 (− 0.837, − 0.222)	0.001
SDS	− 0.202	0.472
CCFI	0.232	0.171
UCEIS	0.032	0.842
*R*^2^	0.537	
Durbin-Watson	1.376	
Defecation urgency threshold		
Fecal calprotectin	− 0.499 (− 0.203, − 0.055)	0.001
Disease duration	− 0.358 (− 1.595, − 0.173)	0.017
IBD-Q	− 0.043	0.803
SAS	0.150	0.374
SDS	0.099	0.552
CCFI	− 0.069	0.624
UCEIS	0.145	0.341
*R*^2^	0.559	
Durbin-Watson	2.033	
Maximum capacity threshold		
Fecal calprotectin	− 0.691 (− 0.360, − 0.108)	0.001
Disease duration	− 0.148 (− 1.576, 0.614)	0.376
IBD-Q	− 0.194 (− 0.665, 0.233)	0.333
CCFI	− 0.125 (− 0.360, − 0.108)	0.437
*R*^2^	0.438	
Durbin-Watson	1.605	

## Discussion

Ulcerative colitis (UC) is a chronic inflammatory bowel disease marked by recurring inflammation, particularly in the rectum [13]. This inflammation leads to intestinal wall fibrosis, resulting in changes in anal and rectal function, such as fecal incontinence [14]. Despite advancements in medications, most UC patients still face significant anorectal limitations, with healing rates of approximately 50%, and some even require surgical intervention [15]. Rao et al. [16] reported differences in anorectal function between patients with active UC and patients in remission. However, there is a lack of studies on impaired anorectal function in recurrent UC patients. Therefore, we conducted a pioneering study to assess changes in anorectal function in this patient group.

In this study, we analyzed the characteristics and anorectal function of 33 patients with recurrent UC and 40 patients with primary UC. Compared with primary UC patients, recurrent UC patients showed rectal hypersensitivity, with lower thresholds for initial sensation, defecation volume, defecation urgency, maximum capacity, and MSP. Moreover, recurrent UC patients had increased levels of hs-CRP and fecal calprotectin, indicating increased inflammation in localized lesions. The modification in rectal sensation may stem from the increased sensitivity of rectal wall receptors due to inflammation. As noted by Rao et al. [17], stretch receptors on the rectal wall are crucial for relaxing the internal anal sphincter during rectal expansion. In the context of mucosal inflammation, these receptors become increasingly sensitized, potentially explaining the observed alterations in patients with recurrent UC.

In addition, our study revealed that compared to patients with primary UC, those with recurrent UC exhibited notably lower BMIs, serum Hb and ALB levels, and IBD-Q scores (all *P* < 0.05). Our findings on the serum ALB and Hb levels in recurrent UC patients were consistent with those of Ishida et al. [18] These authors reported that the average serum ALB concentration was 40.3 ± 4.9 g/L, and the average Hb concentration was 128.7 ± 19.7 g/L in active UC patients, suggesting a shared state of compromised nutritional status and the presence of anemia [18]. Similarly, in our study, the BMI of recurrent UC patients was 20.64 ± 1.76, which is akin to the observation of Farraye et al. [19] of a BMI of 24.6 ± 4.7 in patients with active UC. These findings further indicate the presence of compromised nutritional status [19]. Additionally, the IBD-Q score of recurrent UC patients in our study was 165.3 ± 13.2. This finding parallels the findings of Ghosh et al. [20] that patients with moderate to severe active UC had an average IBD-Q score of 129.0 ± 36.0, thereby underscoring a notable decline in their overall quality of life [20].

Our findings also indicated that recurrent UC patients were more susceptible to fecal incontinence. A meta-analysis by Gu et al. [21] revealed that the overall incidence of fecal incontinence in IBD patients was 37%, and the incidences of incontinence in patients with UC and patients with Crohn’s disease (CD) were comparable. Factors contributing to the increased risk of fecal incontinence in IBD patients include chronic alterations in bowel habits, perianal diseases, loss of the rectal inhibitory reflex, and abnormalities in rectal sensation, among others [21]. In addition, active IBD is associated with the sustained release of inflammatory mediators. This leads to abnormal alterations in gastrointestinal sensorimotor function and visceral hypersensitivity, ultimately contributing to the onset of functional disorders [22]. In our study, the mean anal pressure in recurrent UC patients did not differ from that in primary UC patients, yet inflammatory markers were notably elevated. We thus speculate that fecal incontinence symptoms in recurrent UC patients may result from inflammation-driven abnormal and intense contractions of the rectum, further leading to a reduction in the rectal volume threshold.

Finally, our investigation further revealed that fecal calprotectin levels, anxious state, and disease duration were independent influencing factors for rectal sensitivity in recurrent UC patients. Fecal calprotectin is closely associated with endoscopic disease activity in UC patients. It correlates more strongly with endoscopic activity than with clinical activity and C-reactive protein. Lower fecal calprotectin levels can predict the clinical and endoscopic treatment response in patients with UC​​ [23]. D’Haens et al. [24] reported that fecal calprotectin levels > 250 μg/g have 71.0% sensitivity and 100.0% specificity for detecting active mucosal disease in UC patients (Mayo > 0) and are significantly correlated with UC symptom scores. Furthermore, Van Den Brink et al. [25] reported that among 374 IBD patients (39.6% of whom were UC patients), 35.2% experienced mild anxiety or depression symptoms, and 12.4% experienced severe symptoms, with increased disease activity strongly predicting worsening of depressive symptoms​. In addition, Gracie et al. [26] demonstrated bidirectional influences between peripheral and central factors affecting rectal perception in patients with IBD. Disease activity increased anxiety scores by sixfold, and high anxiety scores increased the risk of flares by threefold [26]. Therefore, worsening anxiety, coupled with chronic inflammation and prolonged disease duration, may diminish rectal sensitivity, affecting anorectal function in recurrent UC patients. This may result from increased inflammatory mediators, rendering intestinal sensory nerves hypersensitive. As inflammation progresses deeper into the intestinal layers, the levels of factors such as vasoactive intestinal peptides and enkephalin decrease in intestinal wall tissues. Clinically, these findings underscore the importance of comprehensive treatment plans that address both the inflammatory and psychological aspects of UC. Integrating psychological support and anxiety management into the treatment regimen could improve rectal sensitivity and overall anorectal function, thereby enhancing the quality of life for recurrent UC patients.

The present study has several limitations. First, the sample size in this investigation was modest. Second, our study cohort comprises referred patients, potentially encompassing individuals with more intricate medical conditions. This circumstance could introduce institutional and selection biases. Third, the study lacked the inclusion of individuals with inactive or severe UC, where rectal compliance and sensitivity may fluctuate with disease remission or activity levels. Finally, no follow-up was conducted to evaluate changes in anorectal function over time in these patients.

## Conclusion

In conclusion, chronic recurrent UC is characterized by increased rectal sensitivity and compromised anorectal function, which notably affect the quality of life of afflicted individuals. Proactive disease management, anxiety mitigation, and relapse reduction constitute efficacious strategies for ameliorating anorectal function in recurrent UC patients.

## Supplementary Information

Below is the link to the electronic supplementary material.

Supplementary file1 (DOCX 17.6 KB)

## Data Availability

The data used in this study are available from the corresponding author upon request.
