# Supplementary material for: Assessing anorectal function in patients with recurrent ulcerative colitis
Source: Int J Colorectal Dis. 2024 Jul 16;39(1):110. doi: 10.1007/s00384-024-04680-1 (PMC11249410; doi:10.1007/s00384-024-04680-1)
Supplement: Supplementary file 1 — Supplementary file1 (DOCX 17.6 KB) [file 384_2024_4680_MOESM1_ESM.docx]

**Supplementary Materials**

**Assessing Anorectal Function in Patients with Recurrent Ulcerative Colitis**

Qiaoyan Wu^1, 2‡^, Tongyu Li^3‡^, Fenglian Deng^1, 2^, Xuejie Yao^1, 2^, Xueqin Chen^4^, Qi Jiang^1, 2*^, Xiaoyun Ding^1, 2*^

^1^Department of Gastroenterology, The First Affiliated Hospital of Ningbo University, No. 59, Liuting Street, Ningbo, 315010, Zhejiang Province, China

^2^Ningbo Key Laboratory of Translational Medicine Research on Gastroenterology and Hepatology, No. 59, Liuting Street, Ningbo, 315010, Zhejiang Province, China

^3^Department of Hematology, The First Affiliated Hospital of Ningbo University, No. 59, Liuting Street, Ningbo, 315010, Zhejiang Province, China

^4^Hospital Quality Management Office, The First Affiliated Hospital of Ningbo University, No. 59, Liuting Street, Ningbo, 315010, Zhejiang Province, China

^‡^Contributed equally.

Correspondence:

Xiaoyun Ding, Ph. D,

E-mail: [dyyyding@126.com](mailto:dyyyding@126.com)

Qi Jiang, MD,

E-mail: jiangqigua@163.com

**Supplementary** **Methods**

**1. Patient enrollment**

Inclusion criteria were as follows: 1) Age between 18 and 70 years; 2) Mild to moderate UC was diagnosed based on clinical signs and specific criteria; 3) Primary UC refers to patients diagnosed for the first time without prior medical intervention, including some who initially opted against seeking treatment; 4) Recurrent UC was characterized by the recurrence of symptoms such as abdominal pain, diarrhea, and bloody stools after a period of remission, often requiring a change in therapy. This recurrence, marked by worsening symptoms and an elevated clinical activity index, includes increased diarrhea and bleeding, persisting over time. Clinical remission was defined as baseline intestinal function without blood. Endoscopic remission was defined as normal endoscopic features, graded as 0 or 1. For initial-onset UC patients, the disease duration was assessed from the time of UC diagnosis to the initiation of medication.

Exclusion criteria included: 1) Severe UC; 2) Recent use of NASIDs, laxatives, prokinetics, or antidepressants within one month, history of diabetes, thyroid disease, pelvic trauma, pregnancy, pelvic radiation, rectal or colon surgery, rectal anal stenosis, rectal adenocarcinoma, anal fissure, fistula, or abscess, and other perianal diseases; 3) Presence of severe cardiovascular, hepatic, pulmonary, cerebral, or malignant conditions that significantly affect quality of life, or inability to comprehend or respond to questionnaire content.

**2. Questionnaire details**

For the SAS scale, these items are rated on a four-level scale according to symptom frequency, including 15 positive items and 5 reverse-scored items. The raw score is multiplied by 1.25 to obtain the standard score, with scores of 50-59 indicating mild anxiety, 60-69 indicating moderate anxiety, and above 69 indicating severe anxiety. Similarly, the SDS scale consists of 20 items, also rated on a four-level scale based on symptom frequency, with 10 positive items and 10 reverse-scored items. The standard score is calculated by multiplying the raw score by 1.25, and scores of 53-62 denote mild depression, 63-72 indicate moderate depression, and above 73 represent severe depression. The Cleveland Clinic fecal incontinence score includes 5 events – solid incontinence, liquid incontinence, gas incontinence, pad use, and lifestyle alteration – to evaluate the severity of fecal incontinence symptoms. Each item is scored from 0 (never) to 4 (always), resulting in a total score range of 0-20, with higher scores indicating more severe fecal incontinence. The UCEIS scoring system assesses the degree of inflammation in ulcerative colitis based on vascular pattern, bleeding, erosion, or ulceration. Scores of 0-1 suggest remission, 2-4 indicate mild inflammation, and 5-8 denote moderate-to-severe inflammation.

**3. Assessment protocol**

Following the standardized procedures outlined in the London Protocol developed by the International Anorectal Physiology Working Group (IAPWG), the following assessments were conducted:

1) Cough Reflex Collection: Instruct the patient to cough. Examine the instant pressure increase in the rectum and anus when abdominal pressure rises during forceful coughing.

2) Resting Pressure Collection: Instruct the patient to relax and rest for 2-5 minutes. Record the resting pressures of the rectum and anus for 30 seconds once the patient has settled into a calm breathing pattern.

3) Guide the patient through movements and collecting corresponding indicators:

a. Anal Contraction Movement: Instruct the patient to forcefully contract the anal sphincter and maintain the contraction as long as possible, up to a tolerance limit of 15 seconds. Perform this sequence three times with at least a 1-minute interval between each.

b. Simulated Defecation Movement: Instruct the patient to simulate a defecation movement and then click "relax" for relaxation. Repeat this sequence three to five times with at least a 1-minute interval between each.

4) Inflating the balloon, inquire about sensations, and collecting corresponding indicators:

Rectoanal Canal Inhibits Reflex (RAIR): Inflate the balloon with air, increasing the volume by 10 mL increments up to 50 mL. Each inflation should be completed within 1-2 seconds, with a quick inflation followed by a rapid deflation, leaving a 30-second interval. Inquire each time whether the patient perceives any sensation.

Evaluating rectal sensitivity: Begin by explaining the four sensations that arise from introducing air into the rectum:

a. Initial Sensory Threshold: The initial feeling of air entering the rectum;

b. Defecation Volume Threshold: The sensation of needing to defecate;

c. Defecation Urgency Threshold: A sense of urgency, albeit still within tolerable limits.

d. Maximum Capacity Threshold: The point at which the sensation becomes nearly intolerable.

5) After performing the aforementioned procedures, further assessments included:

a. Maximum Constrictive Pressure (the forceful contraction pressure of the anal sphincter);

b. Pain Threshold (the volume that triggers discomfort);

c. Rectal Compliance (the rectum's ability to stretch);

d. Minimum Volume to Elicit RAIR (the smallest volume to induce Rectoanal Inhibitory Reflex);

**Reference:**

1. Magro, F., et al., *Third European Evidence-based Consensus on Diagnosis and Management of Ulcerative Colitis. Part 1: Definitions, Diagnosis, Extra-intestinal Manifestations, Pregnancy, Cancer Surveillance, Surgery, and Ileo-anal Pouch Disorders.* J Crohns Colitis, 2017. **11**(6): p. 649-670.

2. El-Zimaity, H., et al., *Beyond Neutrophils for Predicting Relapse and Remission in Ulcerative Colitis.* J Crohns Colitis, 2023. **17**(5): p. 767-776.
